# “How about We Give It a Go?”: A Case Study on Supporting Breastfeeding Families in an Australian Child Care Centre

**DOI:** 10.3390/children7110195

**Published:** 2020-10-23

**Authors:** Emma Woolley, Shirley Wyver

**Affiliations:** Macquarie School of Education, Macquarie University, Sydney, NSW 2109, Australia; shirley.wyver@mq.edu.au

**Keywords:** breast feeding, infant, breast milk expression, child care centres, employed women, family work relationship, early childhood education, return to work, fathers, qualitative research

## Abstract

The World Health Organization (WHO) recommends exclusive breastfeeding for six months and continuation of breastfeeding for up to two years. Formal child care has an important role in supporting breastfeeding, as many Australian infants commence care before two years of age. Currently, little is known about support or barriers in child care contexts. The present qualitative instrumental case study explores practices which protect, promote and support breastfeeding at a child care centre located in the Australian Capital Territory’s outer suburbs. Extending from a previously published collective case study, a cultural-institutional focus of analysis was used to explore the roles of proximity, flexibility and communication in supporting breastfeeding within a child care centre located close to an infant’s home. Interviews with centre staff and mothers, triangulated with observations of the centre environment and policy documents provide insight into the environment. Affirming the roles of flexibility in routine and staff rostering and two-way communication, findings suggest longer-term benefits may be derived from selecting a child care centre close to an infant’s home, provided mothers can overcome barriers to breastmilk expression in the workplace. The study recognises the role of non-lactating caregivers in the transition to formal child care, and of the support culture for educators who breastfeed. This study extends the knowledge base of breastfeeding support interventions in the child care setting to inform future research and policy.

## 1. Introduction

There is strong evidence for the health, societal and economic benefits of the protection, promotion and support for breastfeeding, for both mother and child [1,2]. Infants who are not breastfed have a higher risk of developing respiratory illnesses, asthma, otitis media, and diet-related chronic disease in later life [1]. Health risks for mothers who do not breastfeed include a slower recovery from childbirth, higher rates of maternal depression, and higher rates of breast and ovarian cancer [1]. Breastfeeding is also thought to have a protective effect against Sudden Infant Death Syndrome (SIDS) [3]. Economic benefits to breastfeeding include lower net food costs to households, and lower overall health care costs [4].

The World Health Organization (WHO) recommends exclusive breastfeeding until around six months of age, complemented by family foods until 24 months of age and beyond [5]. WHO also notes that mothers need support for breastfeeding to be initiated and sustained [6]. Many countries, including Australia, have developed guidelines that align with WHO recommendations but include variations that may be more appropriate for the local context [6]. Australia’s Infant Feeding Guidelines maintain the WHO recommendation of exclusive breastfeeding in the first six months, but then emphasise 12 months of age and beyond for continuation of breastfeeding [7]. Commensurate with many other high-income countries, Australia has high rates of initiation of breastfeeding, but low duration rates, with only 12% of mother-infant dyads achieving the National Health and Medical Research Council (NHMRC) benchmark of receiving any breastmilk at 12 months of age, and 7% of dyads achieving the WHO benchmark of receiving any breastmilk at 24 months of age [8].

In Australia, a mother’s return to work is often cited as a contributing factor in the cessation of breastfeeding [8]. Formal child care is an important enabler of mothers’ workforce participation in Australia [9]. Australian settings-based population health interventions indicate that child care may be well suited to actively supported breastfeeding as well as supporting a mother’s transition back to the workplace [10,11,12]. Although tentative steps have been made towards ‘breastfeeding friendly child care’ programs, researchers have recognised the lack of evidence as a barrier to designing and implementing effective population health interventions to improve breastfeeding rates through the setting [13].

There is a paucity of research on scalable population health interventions to promote, protect and support breastfeeding in Australian child care settings. Research has been limited to several foundational studies, which have identified physical elements of the formal child care environment likely to facilitate breastfeeding, and explored educators’ understanding of anti-discrimination legislation related to breastfeeding [13,14,15,16]. An interdisciplinary case study of two long day care centres situated within a multi-site university campus in Melbourne developed a model to demonstrate the complex interplay of three distinct, but related, communities: the early childhood education and care centre, the mother’s workplace, and the child’s family [17,18]. Within these communities, three interrelated factors impacted the dyad’s experience: the proximity of the child care centre to the mother’s workplace; reciprocal flexibility of child care educators and families; and two-way communication between staff and mothers.

Rogoff’s [19] three foci of analysis provide a useful method for ‘making sense’ of the intersecting elements of breastfeeding support. Cultural institutions examined by Monk et al. [17] were the child care centre, the family and, to a lesser extent, the mother’s workplace. Particularly within the domain of communication, considering the individual and interpersonal aspects of cultural-institutional practices remained essential to understanding breastfeeding as a cultural practice. The use of an instrumental case study methodology is well suited to understanding the intersecting elements of support offered by a child care centre, providing insight into the complexity of the centre’s practice and allowing refinement of the theory presented by Monk et al. [17].

Rogoff [19] describes breastfeeding as a natural practice, with significant cultural differences in the ways in which it occurs across the world. Infants, their mothers, other caregivers and their broader community create the conditions and circumstances in which breastfeeding occurs. In Australia, segregation of infants and their mothers on a mother’s return to work is expected. Rogoff’s [19] foci of analysis model offers a way of examining this complexity. Rogoff’s three analytical lenses can be used to describe the cultural-institutional expectations, the interpersonal interactions and the individual choices related to infant feeding. Rogoff applied the approach to studies of mother-infant relationships in the context of sleep and bed-sharing, and it is well-suited to research in child care settings, as it considers the multiple influences on a child’s development within a complex system [17,20]. There are multiple cultural institutions which influence successful breastfeeding, including the infant’s family, the child care centre, and the mother’s workplace [17].

Our study aimed to extend and elaborate on the existing literature, in particular, the work of Monk et al. [17], by applying their methodology to a child care centre that is not co-located with a university or other workplace, to facilitate further examination of the importance of proximity between a mother’s workplace and her child’s child care centre. In addition, we sought to document and understand further influences that may impact breastfeeding support provided in child care. We were interested in the interaction between factors within a system rather the presence or absence of particular features. We therefore chose to conduct a detailed case study of a single child care centre. It is impossible to identify a typical child care centre in Australia, but we ensured we selected a centre type and location that was not atypical. Most Australians live in major cities (including surrounding suburbs) and in households with parents and children, but not extended family [21]. Long Day Care is the most frequently used form of child care in Australia and is recommended for parents who work on week days [22,23]. We therefore purposively selected a long day care centre in a major city, Canberra, located in the Australian Capital Territory.

## 2. Materials and Methods

### 2.1. Participants

The selected child care centre was a small owner-operated long day care service located on Canberra’s rural-urban fringe, employing five educators to care for up to 25 children each day. The centre’s philosophy described the value of a home-like environment for holistic child development, and was visible in the centre’s physical environment (a converted house in a residential area).

Four early childhood educators, including the centre’s Director (an early childhood teacher-qualified leader), and two mothers participated in the study. Eligibility criteria were over 18 years of age, sufficient understanding of written and spoken English to enable participation and be an educator who provided care for infants; hold a leadership position at the centre; or be a parent of a child attending the centre who had ever breastfed. The two mothers interviewed were employed in professional roles, around 30 min’ drive from the centre.

### 2.2. Procedure

A qualitative instrumental case study approach was used to provide an in-depth study of the complex phenomenon of breastfeeding attitudes and practices among early childhood educators and families in a purposively selected real-life setting. The study used multiple data collection methods, led by in-depth semi-structured interviews with centre’s management, educators and mothers of infants attending the centre, supported by observations of the centre environment and collection of relevant centre documents. Given the important role of partnerships between families and educators in facilitating infant feeding, multiple perspectives were required to answer the research questions and understand the features of the child care centre that protect, promote and support breastfeeding. All interviews and observations were conducted by the first author and commenced after a series of visits to the centre and conversations with the director to establish rapport and understand the centre philosophy.

The study procedures were approved by the Macquarie University Human Research Ethics Committee’s Human Sciences Subcommittee (Reference No 5201834806742). Data collection was undertaken between March and May 2019. The study included two recruitment procedures. First, directors of child care centres who would be well-placed to assist in answering the research questions were directly approached. Second, centre staff and mothers of children at the centre were invited to participate in an in-person, in-depth semi-structured interview. Written consent was obtained from the Director on behalf of the centre, and also from individual interview participants.

Interviews were held at the child care centre, or at a public place of the participant’s choice. Interviews were conducted by the first author using an interview guide. Educators were invited to share their experiences of supporting breastfed infants, and the support systems that they have developed at their service. Mothers were asked to share their experiences of care at the child care centre, and also their broader experiences of returning to work and support systems. All participants were probed about their views on proximity in particular. The contents of the interviews were not disclosed to other participants, including the Director. Half of the interviews were transcribed using manual transcription, with the remaining interviews transcribed by a paid transcription service. Transcripts were provided to participants for member-checking.

Participants (mothers and child care centre staff) were also asked to complete the Iowa Infant Feeding Attitude Scale (IIFAS), to provide insight into their attitude toward the process and product aspects of breastfeeding [24]. A list of participants, their personal experiences of breastfeeding, and their IIFAS scores are provided in Table 1. Staff participating in the study had mixed personal experiences of breastfeeding. Two educators, including the child care centre’s Director, reported that they had breastfed their own children in line with the Australian recommendations. One educator ceased breastfeeding her first child after experiencing difficulties and used infant formula exclusively with her second child; one educator had no children. IIFAS scores suggested half held positive attitudes towards breastfeeding—although this is based on validation scores for mothers, as the scale has not been validated in this group. Interestingly, it was the two educators who had breastfed their own child who had scores in the positive range. Scores for both mothers indicated a positive attitude towards breastfeeding.

Field notes of observations were made to describe the physical environment of the child care centre, to note contextual information and to note open discussion about the topic. Organisational documentation, such as policies and procedures, orientation information and templates and Assessment and Rating reports were collected.

### 2.3. Data Analysis

The collected data was analysed using a cultural-institutional perspective, with reference to Hedegaard’s [25] common-sense level of interpretation. Common-sense interpretation, as described by Hedegaard [25] (p. 58) is a reflection of the activity setting by the researcher. The results presented in this paper are a combination of common-sense interpretation of the first author, and perspectives of the researched persons, in this case, the child care centre’s leadership, educators and mothers. The second author checked interpretations against the transcripts, documents and field notes collected. Differences in interpretation were resolved by discussion.

Simple patterns in relationships and interactions were identified and described by analysing the interview data, organisational documents and fieldwork notes, in accordance with Hedegaard’s [25] interpretative approach. These patterns were considered using Rogoff’s [19] cultural-institutional focus of analysis on the three elements of proximity, flexibility and communication, to understand their impact on breastfeeding practices within the long day care setting. Rogoff’s [19] three planes of analysis tool was used to analyse these patterns from the perspectives of the educator, the mother and the researcher. Analysis adopted a cultural-institutional focus, while retaining background information from the personal and interpersonal planes. Cultural institutions examined in this study were the child care centre, the family and, to a lesser extent, the mother’s workplace.

## 3. Results

Along with examining the influence of proximity, flexibility and communication on the dyadic breastfeeding relationship, two further influences on breastfeeding protection, promotion and support were identified. These influences were (1) the role of fathers and other non-lactating caregivers in supporting the breastfeeding relationship, particularly during an infant’s transition into formal child care; and (2) the importance of creating breastfeeding-friendly work environments for educators themselves.

### 3.1. Proximity, Flexibility and Communication

Having a child care centre located close to a mother’s workplace wasn’t viewed as particularly important by any participant in this study. With the child care centre located around twenty minutes’ drive from the Australian Capital Territory’s employment centres, having a mother attend the centre during the day to feed her infant directly from the breast was very rare.

*“About twenty-five years ago, we had someone who would leave [her workplace] … and come down … to nurse.” (Director)*

*“I can’t think that we’ve had any come and visit to breastfeed and then leave.” (Educator 1)*

Both mothers interviewed were employed in professional roles, and had flexible working arrangements in place, including the ability to work from home, paid lactation breaks and flexible days and hours of work. Both mothers had selected a child care centre close to their home, so that they could easily use the centre while working from home, or when not at work, and so that their partners could share drop-off and pick-up responsibilities.

*“We both work split shifts … so that way, they’re in daycare shorter.” (Mother 2)*

*“I do pick up most days. His dad does drop-off most days … I don’t want that added rush of trying to get him to daycare.” (Mother 1)*

When prompted about whether this decision impacted her experience of breastfeeding, one mother explained that it made for an easier transition at the end of the day to be close to home.

*“It takes five minutes, you know, just to get him in the car, drive home, get him out of the car, and then I can feed him somewhere more comfortable.” (Mother 1)*

Central to the child care centre’s approach was operating with a high educator-to-child ratio, which facilitated their flexible practices. Educators understood that the transition between home and the care environment could be lengthy and infants were not expected to immediately adapt to institutional practices. All educators described flexibility in the centre’s routine to accommodate infants’ individual feeding and sleep needs.

*“They need to sleep on a person until they’re comfortable to sleep on a bed, so that’s what we do. Whoever their preferred educator is, that person is the child’s bed for that hour, or half hour.” (Director)*

Educators described how above-ratio staffing allowed them to support infants experiencing difficulty with settling, even on busier days, and how it enabled them to focus their attention one-on-one for as long as needed. At the centre, feeding infants on-demand was encouraged, and children were able to sleep and rest on their own schedule. The child care centre employed a range of flexible practices to support this approach, including flexible timing, routines and staff rostering.

*“From a planning perspective, a regular time would definitely be easier, but logically, children don’t necessarily understand time.” (Director)*

*“Wednesdays are busy. I think we’ve got ten babies on a Wednesday, so that can be hectic. But still we manage to hold onto babies if they need to sleep in our arms. If we know we can transition them to the bed, then we do that, but if not, we hold them until they wake up.” (Educator 1)*

Educators attributed consistent communication with families to low staff turnover, and the use of familiar relief educators.

*“I think we’re all familiar faces… there is no turnover of staff.” (Educator 3)*

Educator 3 went on to describe how she communicates her willingness to support mothers to continue to provide breastmilk for their infants, while also respecting the infant feeding choices of individual families, by gently probing mothers about a decision to wean.

*“It’s your choice. How do you feel about it? Is there a reason? How about we give it (offering breastmilk) a go?” (Educator 3)*

In general, educators balanced their encouragement of breastfeeding with a desire to support individual mothers’ choices in how they fed their infants. They managed this by informing families during orientation that they would adapt their practice to meet the individual child’s needs. When prompted to describe the centre’s orientation procedures, Educator 3 explained how mothers were often concerned about burdening educators with additional work when providing expressed breastmilk.

*“I find that parents, they don’t want to put us out. It’s like an embarrassment.” (Educator 3)*

She worked to communicate her willingness to adapt her practice to the individual infant and offered practical examples to mothers of how she could support them to continue to breastfeed, such as adding expressed breastmilk to cereal for older infants, using fathers or alternative caregivers to introduce a bottle or cup and holding infants who fall asleep during their feed for the duration of their nap.

*“I encourage it a wee bit without even being aware I’m actually doing that.” (Educator 3)*

The centre’s parent information handbook encouraged families to spend time at the centre on arrival and departure to promote positive relationships between children, staff and families; and welcomes families to call throughout the day to share information with educators. Educators described how these practices enable educators to provide feedback to families each day about the volume of expressed breastmilk supplied and consumed, and whether any adjustments were needed. This information was vital to mothers, as significant time and effort was required to express the milk.

*“I use my lunch breaks and my tea break to express or feed, then I get an extra 15 min paid break as well, because I need 4 breaks a day.” (Mother 1)*

Mother 2 emphasised that it was important that educators appreciated how much effort she put in to expressing breastmilk while at work, and how her experiences of breastmilk expression were linked to her mental health and wellbeing. She described how she spent all of her break times at work expressing breastmilk, and that she noticed her mental health was declining. To reduce the amount of time she spent expressing, she approached the educators to request daily updates about how much expressed breastmilk her daughter was consuming across the day.

*“I’ve noticed that in the last, say, three weeks, that it’s really affecting my mental health, sitting in an office expressing all day … What I’ve started to do the last couple of weeks is just call daycare, before my last expressing break … because I don’t really like doing it that much.” (Mother 2)*

Improving communication with the child care centre allowed Mother 2 to reduce the amount of expressed breastmilk she was providing and noted an improvement in her mental health once she was able to replace her lunchtime expressing session with a walk outdoors.

*“I’ve started to call them, just to go … ‘Have you given her the bottle?’, so that way I can determine whether I need to express or to hold off.” (Mother 2)*

### 3.2. The Role of Fathers and Other Non-Lactating Caregivers

The involvement of fathers in managing an infant’s transition to care was a recurring theme in participant interviews. Adopting a cultural-institutional focus to examine the transition to formal child care for breastfed infants supported the exploration of relationships beyond the mother-infant dyad and educator relationship. In this study, fathers were defined as a male parent within the context of a parenting partnership; however, the descriptions of their role in supporting the breastfeeding relationship could be expanded to include other caregivers, including a non-lactating parent.

A particular feature of the experience of breastfeeding families at the studied child care centre was the close involvement of fathers in their infant’s transition to formal child care. Both mothers interviewed highlighted the importance of their partner in supporting them to continue breastfeeding on their return to work. Their partner’s support affirmed each mother’s decision to continue to breastfeed.

*“He’s just supportive of it. I think he prefers the breastfeeding because… it’s just easier.” (Mother 2)*

Educators commented that fathers were important figures in helping an infant to accept expressed breastmilk from a bottle or cup while at the child care centre. Educators actively sought information from fathers about how to best introduce a bottle, and their infant’s preferences and routines.

*“[I asked,] ‘does Dad feed the baby when you’re tired?’ So Dad goes, ‘this is how I do it, and that’s how I hold him.’” (Educator 3)*

*“I think dads are expected to provide expressed breastmilk or a bottle, aren’t they? They are the ones that do it. It’s not always Mum.” (Educator 3)*

One educator gave an example of an infant who was experiencing difficulty in adjusting to being fed from a bottle in their early days at the child care centre.

*“We had one little boy who, I would say, the first two weeks, just cried and wouldn’t take a bottle from us.” (Educator 1)*

The educators communicated their concerns with the family. On receiving this information, the family suggested that having the mother to attend the child care centre may result on further stress for the infant on her subsequent departure, possibly resulting in the infant leaving care for the day. Instead, the infant’s father attended the centre to support educators to give the bottle and would leave once the child was settled.

*“Mum didn’t want to interfere with that, because then the baby would have wanted to breastfeed, and probably just go home.” (Educator 1)*

*“He was great, too. When we rang him up and said such-and-such, he just hasn’t drunk or hasn’t eaten, he’d come in within 15 min, give him his bottle and leave.” (Educator 1)*

### 3.3. Breastfeeding Friendly Work Environments for Educators

The studied child care centre’s approach to flexible care was rooted in the Director’s first-hand experience of feeding her infant while working at the centre. The Director had returned to the centre after a short period of maternity leave and looked after both her children as infants at the centre.

*“I nursed both my children here.” (Director)*

All participants indicated that the breastfeeding-friendly culture at the centre was facilitated by the Director, and informed by her personal and professional experience, values and deeply held beliefs. The Director set clear expectations to staff and families that discrimination of breastfeeding mothers would not be accepted.

*“I was very confident and comfortable in my breastfeeding, and my right to breastfeed, so it was not something that was ever questioned.” (Director)*

While proximity between workplace and child care centre was not seen as essential for families, the role of reducing separation between mother and infant to facilitate breastfeeding was acknowledged in the support available to staff who breastfed. This was demonstrated by allowing educators to enrol their own children at the centre, or to have their infants brought to them to breastfeed. More broadly, the family-oriented work environment of the allowed educators who are parents to also attend to their caring responsibilities.

*“The whole reason we opened it up to babies was so that the staff, if they were to fall pregnant and have babies, could have their babies here with them.” (Director)*

Educators were provided with both the physical facilities, and flexibility in rostering to breastfeed or express for their own infants. Flexible room groupings and high educator-to-child ratio meant that supervision was able to be maintained while a breastfeeding educator was feeding her infant, and staff assisted each other.

*“If she was going to breastfeed … obviously we wouldn’t be expecting her to be able to get up and do things, so there would always be one or two other staff members nearby to help.” (Director)*

Informal arrangements were also used by the child care centre to support educators who are parents to attend to family responsibilities. Where a breastfeeding educator did not have their child enrolled at the centre, they were encouraged to have their infant’s caregiver bring their infant in to feed at any time. Telephone access was readily available for educators to facilitate this.

*“One of our other teachers lived close by, and while her husband was off, he would bring the baby in for a breastfeed, or she would go and express.” (Director)*

The child care centre lacked formal policies documenting their approach; however all employees were clear, when prompted, that they would feel supported to breastfeed while working at the centre. While not all educators shared the Director’s openly positive attitude to breastfeeding, they were all willing to accommodate breastfed infants. One educator, who had experienced difficulties in breastfeeding her own children, conveyed the tension she felt between her personal feeding experiences, and her professional role as an early childhood teacher.

*“I don’t think it’s important, but I think when it happens, it should be freely spoken about. It’s not a taboo subject here.” (Educator 1)*

*“If they come in and say that they are breastfeeding, we say, yes, okay, you tell us what you need to do.” (Educator 1)*

## 4. Discussion

At the heart of the studied child care centre’s approach to supporting infants was high educator-to-child ratios within a mixed aged group setting, and a firm understanding of the practicalities of feeding infants on-demand. Working above mandated ratio requirements allowed educators to have more one-on-one time with individual infants, with an informal primary caregiver approach adopted at times. Supporting one-on-one time between infant and educator allowed each infant’s individual routine, including arrival and departure, feeding, sleeping, and changing, to be conducted uninterrupted, promoting trust and attachment.

In place of formal training, educators primarily reflected on the philosophy of the child care centre in determining how to best offer infant feeding support to families. This was more important than public health resources designed to promote breast feeding in child care centres. The approach developed within the centre had clear benefits of relating directly to the centre resources and provided flexibility to accommodate infants, parents and educators. At the same time, the vulnerabilities were apparent. For the participants in this study, educator attitudes to breastfeeding, based on the IIFAS, seemed to be associated with experience of being a lactating parent. The educators’ contrasting personal experiences of breastfeeding, as discussed during interviews, illustrates potential difficulties in developing a ”breastfeeding-friendly” culture and establishing a consensus on how a child care centre can best support a family to continue to provide breastmilk for their infant. In turn, working towards a shared understanding of individual families’ breastfeeding goals may be difficult.

The disconnect between public health documents designed to promote breastfeeding friendly environments and the development of practices within this case study is worthy of further research. Clearly, educators want to support the decisions of all parents and specific promotion of breastfeeding therefore presents a dilemma. Current resources were perceived as being too ‘pro-breastfeeding’ and potentially distressing to mothers who do not breastfeed. The child care centre in this study had low/no turnover (as mentioned by Educator 3), but the sector generally has very high turnover with an estimate of 80% turnover every six years [26]. While the child care centre in the present study relied heavily on their philosophy and team communication, it seems unlikely that this would be enough for child care centres with higher levels of turnover and greater external support may be needed. Larger scale studies are required to further understand the links between child care centre philosophies, formal training and breastfeeding promotion resources.

The child care centre’s policies emphasised the importance of developing meaningful relationships with families, however, a gap between policy and practice on development of shared understanding between the mother and the educators was evident. Both mothers had mutual agreement of their breastfeeding goals with their partner, but not with the child care centre. Both mothers worked with their partners to undertake actions that would support them to maintain their breastmilk supply and manage their return to work. With their partner, they regularly revisited their breastfeeding goals, and actively collaborated to work towards them, negotiating transitions and workload. The same level of participation and collaboration was not evident in the mother’s relationship with child care centre staff, although there was mutual acknowledgement of a broad goal to continue to breastfeed.

Facilitating breastfeeding-friendly working environments for early childhood educators may be the key to creating a broader breastfeeding-friendly culture in the sector. A lack of formal training or professional development for early childhood educators on breastfeeding topics meant that educators relied on peer-to-peer learning to develop their knowledge of providing expressed breastmilk to infants and supporting families. This reliance on peer-sharing may mean that the breastfeeding experiences of educators themselves are a major contributor to the breastfeeding support culture of individual child care centres. Support for educators who breastfeed may be a key component of developing a breastfeeding-friendly culture within the child care sector.

Previous studies have described the value of co-locating child care centres with workplaces to promote and protect the breastfeeding relationship between mother and infant within some families [16,27]. When feeding directly from the breast, co-location reduces the amount of time a mother is separated from her infant. However, the mothers interviewed in this study were able to successfully breastfeed their infants using expressed breastmilk on their return to work. The benefits of proximity between the child care centre and the family’s home and the father’s workplace were highlighted by participants.

The culture and attitudes of mothers’ workplaces significantly influenced their expectations of their ability to combine breastfeeding and formal child care. Both mothers in the present study worked in the public service, and they both expected that their workplace would offer flexible conditions. Established family-friendly practices at each of the interviewed mothers’ workplaces facilitated their continued provision of breastmilk to their infants, through regular breastmilk expression. Enabling factors for the mothers interviewed included paid lactation breaks, dedicated breastmilk expressing facilities, and flexible working conditions, such as an option to work from home or flexible scheduling. Both mothers interviewed had access to flexible work scheduling for both themselves and their partner, which enabled them to select work commencement and departure times within a range set by their employer and reduce the number of hours their infant was away from their family.

If a mother can overcome workplace barriers to expressing breastmilk, and not need to feed directly from the breast, a wider choice of child care centres may be available to her. Other benefits may be drawn from choosing a centre closer to the family’s home, such as the involvement of the child’s father or other caregivers, and longer-term relationships within the community. The length of time an infant is dependent on either breastmilk or infant formula is relatively short in the wider context of a centre-family relationship that may extend to the child commencing school.

The strength of this current study is that it was able to gain insight from breastfeeding mothers who had selected a child care centre for reasons other than proximity to their workplace. The mothers’ identified other priorities in selecting a child care centre, with a focus on their long-term relationship with the centre, and minimising the risk of discontinuous care due to a change in employment circumstances. While these findings differ from the identified previous research on breastfeeding and child care research in Australia, they are consistent with other studies describing parent’s reasons for choice of formal child care setting [28,29,30]. Post-COVID-19, as permanent or regular working from home arrangements become more commonplace, the place of proximity between an infant’s child care centre and their mother’s workplace in facilitating breastfeeding may require re-examination.

The participants in this study echoed previous research highlighting the importance of effective two-way communication between the child care centre and the family [17]. Both educators and mothers emphasised that communication on infant feeding needed to be individualised and that each family was different in their communication needs. A perpetual health promotion dilemma for educators is that they are balancing their roles as advocates for children’s health and wellbeing with their responsibility to respect decisions made by parents as a child’s primary caregiver. The careful language used by staff at families’ first contact with the centre sought to help mothers seeking to enrol in the child care centre to understand that they would be supported to continue breastfeeding, but that their individual feeding choices would also be respected. This careful balance of advocacy, support and respect continued throughout the educators’ relationship with the family through orientation and beyond, but perhaps inhibited active participation towards a mutual goal.

Support measures at the studied child care centre were largely initiated by mothers. With reference to Rogoff’s [19] concept of guided participation, facilitating or limiting access to information on breastfeeding at the centre may influence whether the topic is raised at first contact. Both mothers who participated in the study were motivated to continue breastfeeding on their return to work, and initially raised the topic of infant feeding with the Director at orientation. It is not clear from the findings how a mother with less support from her partner, or who was equivocal about breastfeeding would be motivated by the centre to continue breastfeeding on her return to work. A lack of dedicated policy, promotional material, and specific information in orientation materials may not prompt a mother to raise the topic of infant feeding on initial contact with the child care centre.

Short conversations at the end of each day with the rostered educator, supplemented by occasional phone calls during the day, aimed to assist mothers to understand their infant’s milk intake, and to plan their provision of expressed breastmilk accordingly. In general, the depth of conversation with mothers remained at a procedural level, rather than collaborative. Proactive day-to-day communication from the centre regarding their infant’s expressed breastmilk intake was valued by mothers, as it helped them to plan their expression breaks at work. Reducing the amount of time spent expressing breastmilk was a common goal between the mothers and achieving it may have a positive impact on mothers’ mental health and broader experiences of returning to work. Further collaboration between the mother and the child care centre, such as coordinating the introduction of solid food, or working together to improve start- and end-of-day transitions, may enhance shared understanding.

An unanticipated but important finding was that fathers modelled for educators how to offer a bottle to their infant, were on-call to assist with difficulties with feeding and settling and took part in the transitions and routines at the beginning- and end-of-day. This suggests that the use of a child care centre close to an infant’s home, rather than the mother’s workplace, may promote father-inclusive practice. Given the significant influence that father may have on a mother’s decision to initiate and continue breastfeeding, the involvement of fathers in supporting their breastfed infant’s transition to a formal child care environment requires further exploration.

The Australian child care workforce is becoming increasingly casualised, suggesting educators have limited access to paid maternity leave and lactation breaks, which are both factors associated with improving breastfeeding rates [31,32]. Overcoming workplace barriers to breastfeeding and breastmilk expression for early childhood educators may have a positive impact on the development of a breastfeeding-friendly culture across the sector by improving knowledge about practical support for breastfeeding mothers and infants.

A limitation of both this study, and the previous study [17], is that the mothers interviewed widely relied on support from their family. Future research may improve understanding about how mothers without partners or family members nearby value proximity between their workplace and the child care centre. Other limitations of this study include those common to case study research, including an inability to generalize results to larger populations. Similarly, in comparing the educator’s interview responses to their responses to the IIFAS questionnaire, the IIFAS scores and interview responses appeared consistent; however, the tool provided limited other insights. Further validation of the tool is required to better establish its validity in child care contexts.

## 5. Conclusions

In conclusion, though proximity between a child care centre and a mother’s workplace is thought to be a protective factor for breastfeeding, consideration should also be given to proximity between the child care centre and both the family’s home and the non-lactating parent or other caregiver’s workplace. Collaboration between educators and families, including the involvement of fathers supports working towards a shared understanding or common goal of prolonging breastfeeding.

Broadly, this study suggests that the involvement of fathers in orientation to the setting in this practical, meaningful way may support a successful transition for an infant who is fed expressed-breastmilk. In turn, this may support fathers’ familiarity and comfort with the setting, leading to improved future engagement.

Finally, given the nature of informal peer-sharing of knowledge of breastfeeding support strategies, the development of a breastfeeding-friendly culture across the formal child care sector may be linked to improving breastfeeding rates among educators themselves. Opportunities for improving working conditions for educators may include increasing access to paid lactation breaks, offering flexible scheduling and providing space and support for educators to breastfeed their own infants or express breastmilk.

## Figures and Tables

**Table 1:** Participant characteristics.

Participant	Own Breastfeeding Experience	IIFAS Score
Director	Breastfed own child	65
Educator 1	Mixed breastfed first child, formula fed second child	52
Educator 2	No experience of breastfeeding a child	53
Educator 3	Breastfed own child	59
Mother 1	Breastfed own child, supplemented with formula as needed	66
Mother 2	Mixed breastfed first child, breastfed second child	59

IIFAS: Iowa Infant Feeding Attitude Scale.
